# Resilience and related factors as predictors of relapse risk in patients with substance use disorder: a cross-sectional study

**DOI:** 10.1186/s13011-021-00377-8

**Published:** 2021-05-04

**Authors:** Ayako Yamashita, Shin-ichi Yoshioka, Yuki Yajima

**Affiliations:** 1 Faculty of Medicine, School of Health Sciences, Kagoshima University, 8-35-1 Sakuragaoka, Kagoshima, 890-8544 Japan; 2 Faculty of Medicine, School of Health Science, Tottori University, Yonago, Japan; 3 Department of Nursing, Faculty of Human Health Sciences, Niimi University, Niimi, Japan

**Keywords:** Resilience, Recurrence, Substance use disorder, Employment, Recovery

## Abstract

**Background:**

Resilience, referring to the inherent ability to naturally recover in the face of adverse conditions, is an essential concept in discussions of substance use disorder (SUD) recovery. This study’s objective was to shed light on resilience and related factors that affect relapse risk in patients with SUDs.

**Method:**

Fifty-two patients with SUDs were given a self-administrated questionnaire from February to April 2015 consisting of question items for sociodemographic characteristics, relapse risk (Stimulant Relapse Risk Scale), and resilience (Bidimensional Resilience Scale). Scale scores were tested for associations with subject attributes, after which resilience’s effects on relapse risk were analyzed using correlation and multiple regression (forced-entry) analyses.

**Results:**

Stimulants were the most common substance related to SUD (*n* = 26, 21.7%; multiple answers). Bivariate correlation showed that higher acquired resilience was significantly associated with a lower relapse risk (*r* = − 0.314, *P* < 0.01). Reduced relapse risk was significantly associated with current employment (Std. β = − 0.446, *P* < 0.05).

**Conclusion:**

Our findings demonstrate the necessity of recovery support to enhance acquired resistance in patients with SUDs to prevent relapses. Reinforcing employment support services and encouraging patients to continue treatment were suggested as potentially effective measures to enhance resilience in individuals with SUDs on their road to recovery.

## Introduction

Substance use disorders (SUDs) are a group of mental illnesses characterized by impulsive behavior and a persistent, strong desire to ingest a given substance that are associated with drug-seeking behavior, withdrawal symptoms, and tolerance [1].

SUDs can involve a wide variety of drugs, including stimulants, analgesics, hypnotics, and narcotics [2]; potential for mental and/or physical dependence depends in part on the substance’s mechanism of action. According to 2017 estimates, 2.3% of Japan’s population have experience a SUD at least once in their lives. Cannabis is the most commonly used substance, with an estimated lifetime prevalence of 1.4% [3]. According to a 2016 fact-finding survey regarding the number of patients with SUDs in Japan [4], among the 2262 patients either hospitalized or receiving outpatient treatment at psychiatric facilities, 1209 (53.4%) were addicted to amphetamines, followed by 384 (17.0%) that were addicted to sleeping pills or tranquilizers. Thus, in psychiatric treatment settings in Japan, the principal issue is drug-related disorders related to amphetamine use. However, some commentators have called for an urgent overhaul of Japan’s SUD treatment system, noting the paucity of facilities dedicated to drug rehabilitation and the resulting low rates of treatment among those who need it [5].

Studies have raised cognitive–behavioral therapy (CBT) and stage-transition models as effective interventions for drug dependence [6] and demonstrated the importance of self-awareness and its association with drug cessation [7]. Coping skills, recreational activities, and social support have been reported to effectively encourage continued abstinence [8].

Drug dependence can be considered a chronic disease based on its pathology and symptoms, meaning patients with SUDs should be provided with continuous therapy and support even after they have stopped taking the offending substance. Moreover, since denial is a characteristic symptom of drug dependence [9], it is imperative for patients to gain self-management skills by deeply understanding their disease and personally feeling the benefits of recovery.

Resilience is a concept that has attracted attention in recent years in discussions about recovery from substance dependence [10]. Resilience is the ability to adapt to adverse conditions [11–13], and it is regarded in the field of psychiatry as a critical component of recovery. Resilience does not reflect personal weakness; it can be enhanced in anyone and everyone [14]. Resilience can be subcategorized into *innate resilience*, a product of inherent, individual factors, and *acquired resilience*, which can be modified and strengthened by a variety of environmental factors [15, 16].

Some SUD treatment approaches are based on self-medication theory [17], which hypothesizes that one of the main motives for using illegal substances is that they offer potential temporary relief from psychological distress stemming from chronic sensory overload [18]. The most recent learning and habit theories of addiction posit that drug addiction can be understood in terms of normal learning and memory neuronal systems, which, through chronically self-administered drug actions, are pathologically subverted, thereby leading to the establishment of compulsive drug-seeking habits [19]. Furthermore, because the use of illicit drugs can seriously disrupt a person’s self-control and self-efficacy, it is important to provide people with SUD with support that can increase their resilience and self-esteem [20]. For this population, social support and resilience have been shown to mediate the effects of stress on life satisfaction [21].

Participation in recovery group meetings held in self-help groups and treatment centers helps with SUD by improving self-disclosure [22]. Self-help group participation can constitute a “spiritual” experience, enhancing self-disclosure [23] and allowing attendees to recognize their own experiences in the stories of others [24]. This strengthens resilience, a crucial psychological trait for preventing relapse. This trait is crucial to the concept of recovery as it relates to people with mental disorders. Recovery has been defined as “a process that allows people (with diseases or disabilities) to actively participate in their life, work, study, and community; affected individuals may conceptualize it as the ability to lead a rich and productive life despite their disability, whereas others may see it as a reduction or mitigation of those individuals’ symptoms” [25]. While individual factors certainly play a role in SUD recovery, it is acquired resilience-related factors that are predicted to strongly drive recovery, helping people to grow and develop as they combat their disease.

It has been shown that, to prevent relapse in people with alcohol use disorders (AUDs), attending group meetings sponsored by self-help groups and treatment facilities can help individuals attain deeper levels of self-disclosure, which has been associated with greater resilience [26]. That should also apply in the case of SUDs. Although it is known from previous studies that resilience needs to be increased to prevent SUD relapse, the relationship between resilience and the relapse risk remains unclear.

The purpose of this study was to test the hypothesis that if resilience improved in patients with SUDs who attended a self-help group, their relapse risk would be lower. Showing how resilience may be related to the relapse risk should lead to better recovery support for patients with SUDs.

## Methods

### Study design

This study used a cross-sectional design.

### Study sample

The managers of three drug addiction rehabilitation centers and one medical center specializing in the treatment of substance dependence that sponsored self-help support groups in the Chugoku or Kinki regions of Japan agreed to participate in the study after receiving both verbal and written explanations of the study. The sample consisted of drug-dependent patients attending self-help support groups at these facilities and who consented to participate. Self-help groups had a facilitator trained in evidence-based treatments.

### Data collection

Data were collected using an anonymous self-administered questionnaire. Each facility was asked to distribute and collect the questionnaires and to determine who should be asked to participate. The study took place from February through March 2015. The facilities were provided with 80 questionnaires. After completing the survey, respondents put the questionnaire in a sealed envelope that they deposited in a collection box set up at each facility. One month later, representatives at each facility mailed the surveys to the researchers. A total of 52 responses were received (response rate: 65%).

### Demographics

Demographic features asked about in the questionnaire were as follows: age, gender, whether they lived with another person or had a key person with whom to consult in their lives, employment status, age when diagnosed with SUD, treatment time period (estimated lifetime total), abstinence period, the number of sessions and time period participating in self-help groups, the types of dependence drugs, other psychological disorders, and physical disorders.

### Resilience

This study used the Bidimensional Resilience Scale developed in Japan [27, 28]. This scale comprises innate resilience factors (12 questions related to individual factors) and acquired resilience factors (9 questions related to environmental factors). The innate resilience factors indicate those that are strongly related to the individual’s inherent nature; acquired factors indicate learned methods of resilience. The innate resilience factors included optimism, control, sociability, and vitality; acquired resilience factors included attempting to solve a problem, self-understanding, and understanding others. This scale uses a five-point rating for the questionnaire, with higher total scores indicating greater resilience. In this study, the Cronbach’s α coefficient for the Bidimensional Resilience Scale was 0.797; thus, internal consistency was maintained.

### Stimulant relapse risk

To measure the risk of SUD relapse, we used the Stimulant Relapse Risk Scale (SRRS) [29]. This scale consists of 30 items on 5 subscales as follows: anxiety and intention to use drugs (AI); emotionality problem (EP); compulsivity for drugs (CD); positive expectancies and lack of control over drugs (PL); and lack of negative expectancy for drugs (NE). The SRRS includes five items to measure insight into mental condition: awareness of illness (AI). Examples of the items are “The feeling I used to have while using the drug sometimes comes back” (AI); “I feel a constant need to put something in my mouth” (EP); “I would do almost anything in order to use the drug” (CD); “I would use the drug if I were alone” (PL); and “I would not be able to control myself if I use the drug” (NE). Higher average scores for total and subscale items indicate higher relapse risk, and this study only used the total score. All items ask about a drug-related situation in the past 1 week. When 5 supplementary items indicating the respondent’s intensity of awareness of their illness were included, this scale consisted of a total of 35 items. Questions used a three-step rating scale, with higher scores indicating a greater risk of using the drug of dependence. In this study, the Cronbach’s α coefficient for the 35 items in the SRRS was 0.883; thus, internal consistency was maintained.

### Statistical analysis

First, resilience and SRRS score distributions were tested for normality using the Shapiro–Wilk test. Resilience and relapse risk were then compared between subgroups with different demographic characteristics using the Student’s t-test. Next, Pearson product correlation coefficients were calculated among the following variables: current age, age at diagnosis, treatment time period, abstinence period, acquired resilience score, innate resilience score, and SRRS total score. Finally, multiple linear regression analysis via the forced-entry procedure was performed using relapse risk as the dependent variable, innate and acquired resilience as independent variables, and the following control variables: current age, employment status (dummy variable: 1 = employed, 0 = unemployed), age at diagnosis, the treatment time period, and the abstinence period. Variance inflation factors were calculated for each independent variable to check for collinearity in multiple regression analysis: each measured < 4.0, indicating minimal multicollinearity. Given the small sample size and considering the nature of the concept of resilience, comorbid mental illness was not included as a control variable. Statistical analyses were performed using IBM SPSS 24.0 J for Windows (SPSS, Chicago, IL), and the significance level was set at 5% for all tests.

### Ethical considerations

After receiving approval from the Tottori University Faculty of Medicine Ethics Review Committee (approval number 2646), this study was conducted in accordance with the fundamental principles set forth in the Helsinki Declaration. Informed consent was obtained from each participant after the procedure(s) had been fully explained.

## Results

### Subject attributes

Surveys were given to 80 individuals who attended self-help support groups at three drug addiction rehabilitation centers and one medical center specializing in the treatment of substance dependence. Surveys were collected from 52 of those individuals (response rate: 65.0%). Table 1 details their demographics and other attributes.

**Table 1:** Demographic characteristics of the sample

Items	Values	mean ± SD, (Range)
Number of participants	52	
Gender (% male)	88.5	
Age (years), (Range)		37.2 ± 10.0, (20–66)
Employment status (% work)	32.7	
Presence of key person (% yes)	94.2	
Living with someone (% yes)	86.5	
Treatment state (n)		
Outpatient	37	
Inpatient	1	
Untreated	4	
Cessation of treatment	3	
Unknown	7	
Age when diagnosed with SUD (years), (Range)		29.3 ± 9.4, (15–52)
Treatment time period (months), (Range)		67.0 ± 68.9, (5–282)
Abstinence period (months), (Range)		31.9 ± 30.2, (0–132)
Number of sessions and time period participating in self-help groups (weekly)		5.7 ± 2.0
Comorbid mental illness (n)		14
Physical disorders (n)		14

*n* number of participants

Table 2 shows the population’s drugs of dependence. Stimulants were the most common substance involved in SUD, and they were taken by 26 subjects (21.7%; multiple answers).

**Table 2:** Types of drugs of abuse among subjects

Items	n	%
Stimulant	26	21.7
Alcohol	18	15.0
Prescription drugs	21	17.5
Thinner	13	10.8
Others	16	13.3
Synthetic cannabinoid	12	10.0
Cannabis	9	7.5
Opioid	1	0.8
Cocaine	1	0.8
Methylenedioxymethamphetamine	1	0.8
Marijuana	1	0.8
Lysergic acid diethylamide	1	0.8

Multiple answers

*n* number of participants

The study population was actively and continuously participating in treatment: 73.1% were visiting hospitals or receiving inpatient treatment for SUDs, while the complete sample attended meetings in SUD rehabilitation centers or self-help groups 5.7 ± 2.0 times per week on average. In addition, approximately 90% were living with someone else or had someone with whom they could talk about their SUD. The average abstinence period was 2 years 8 months. These statistics suggest that our population consisted of individuals receiving continuing treatment for SUDs who maintained good interpersonal relationships and had some form of social support.

The fact that subjects were abstinent for only 2 years 8 months on average despite receiving treatment for approximately 6 years suggests that people with SUDs require a long time to break away from their disease.

### Scale scores

Table 3 presents resilience and relapse risk scale data.

**Table 3:** Average scale scores

		(*n* = 52)
Items	mean ± SD	(Range)
Bidimensional Resilience Scale		
Innate resilience	35.3 ± 7.3	(23–58)
Acquired resilience	29.4 ± 4.7	(20–41)
Stimulant Relapse Risk Scale		
Anxiety and intention to use drugs	14.5 ± 4.0	(8–23)
Emotionality problem	16.5 ± 4.2	(8–24)
Compulsivity for drugs	5.5 ± 2.1	(4–12)
Positive expectancies and lack of control over drugs	13.0 ± 3.6	(6–8)
Lack of negative expectancy for the drug	5.7 ± 1.9	(4–10)
Awareness of illness	11.9 ± 2.3	(6–15)
Total	67.1 ± 16.5	(43–93)

*n* number of participants

### Attributes versus resilience and SRRS scores

Table 4 contains the results of statistical comparisons between attributes and instrument scores. Relapse risk was significantly lower in employed subjects (t [43] = 2.976, *P* < 0.01) and those with a comorbid mental illness (t [39] = 2.083, *P* < 0.05).

**Table 4:** Comparison of attributes resilience scores and relapse risk score

							(*n* = 52)
Items		Bidimensional Resilience Scale				Stimulant Relapse Risk Scale	
	n	Innate resilience factors		Acquired resilience factors			
		mean ± SD	*P* value	mean ± SD	*P* value	mean ± SD	*P* value
Sex							
Male	46	35.7 ± 7.5	0.442	29.3 ± 4.6	0.612	66.8 ± 12.4	0.645
Female	6	33.2 ± 5.3		30.3 ± 5.2		69. 6 ± 14.4	
Employment status							
Yes	17	37.1 ± 7.1	0.287	29.1 ± 4.7	0.754	60.3 ± 9.7	0.005**
No	35	34.6 ± 7.3		29.6 ± 4.7		70.9 ± 12.4	
Presence of key person							
Yes	49	35.4 ± 7.4	0.988	29.3 ± 4.7	0.218	66.7 ± 12.2	0.762
No	2	35.5 ± 6.4		33.5 ± 0.7		64.0 ± 11.3	
Living with someone							
Yes	45	35.8 ± 7.3	0.255	29.6 ± 4.8	0.498	66.6 ± 12.8	0.443
No	7	31.8 ± 6.6		28.3 ± 4.2		70.8 ± 10.4	
Treatment state							
Under medical treatment	38	35.7 ± 7.6	0.348	29.2 ± 4.6	0.492	67.1 ± 13.8	0.751
Untreated and cessation of treatment	7	34.0 ± 0.0		27.0 ± 0.0		64.0 ± 0.0	
Comorbid mental illness							
Yes	14	37.2 ± 8.2	0.375	29.4 ± 4.2	0.946	60.8 ± 14.0	0.044*
No	33	34.9 ± 7.2		29.5 ± 5.0		68.8 ± 10.2	
Physical disorders							
Yes	14	36.1 ± 6.9	0.795	28.4 ± 3.8	0.431	70.0 ± 14.7	0.430
No	33	35.6 ± 7.6		29.8 ± 4.9		65.8 ± 11.5	

*n* number of participants

Statistical evaluation is performed by the Student’s t-test. *P < 0.5, **P < 0.01

We have used a pairwise method, so the total number is different

### Correlation analysis

Pearson correlation coefficients were calculated among the following variables: current age, age at diagnosis, treatment time period, abstinence period, and total scores for each scale (Table 5). Current age positively and significantly correlated with age at diagnosis (*r* = 0.600, *P* < 0.01) and treatment time period (*r* = 0.492, *P* < 0.01). In addition, treatment time period positively and significantly correlated with the abstinence period (*r* = 0.367, *P* < 0.01). Innate and acquired resilience exhibited a significant correlation as well (*r* = 0.585, *P* < 0.01). The total SRRS score was significantly and negatively correlated with both innate resilience (*r* = − 0.310, *P* < 0.01) and acquired resilience (*r* = − 0.314, *P* < 0.01).

**Table 5:** Correlation of the variables

							(*n* = 38)
	Stimulant Relapse Risk Scale (total)	Age	Age when diagnosed with SUD (years)	Treatment time period (months)	Abstinence period (months)	Innate resilience	Acquired resilience
Stimulant Relapse Risk Scale (total)	1.000	−0.087	− 0.088	0.057	0.101	−0.310**	−0.314**
Age	− 0.087	1.000	0.600**	0.492**	0.189	−0.040	0.130
Age when diagnosed with SUD (years)	−0.088	0.600	1.000	−0.027	−0.149	0.110	0.123
Treatment time period (months)	0.057	0.492**	−0.027	1.000	0.367**	−0.160	0.145
Abstinence period (months)	0.101	0.189	−0.149	0.367**	1.000	−0.007	−0.180
Innate resilience	−0.310**	−0.040	0.110	−0.160	−0.007	1.000	0.585**
Acquired resilience	−0.314**	0.130	0.123	0.145**	−0.180	0.585**	1.000

*n* number of participants, Pearson’s correlation coefficient

Statistical evaluation is performed by the Pearson correlation coefficient. **P < 0.01

### Relapse risk-related factors

Only patients without missing data for scale items were included in this subanalysis (*n* = 38). Multiple regression (forced-entry) analysis was conducted using SRRS total score as the dependent variable and current age, employment status, age at diagnosis, treatment time period, abstinence period, and acquired resilience as independent variables. The model’s multiple correlation coefficient (R) was 0.562 (coefficient of multiple determination [R^2^] = 0.315) (*P* < 0.05). Table 6 contains the standard partial regression coefficients between SRRS total score and each independent variable. Reduced relapse risk was significantly associated with current employment (Std. β = − 0.446, *P* < 0.05). No significant difference was found for acquired resilience (Std. β = − 0.382, *P* < 0.05).

**Table 6:** Relapse risk factors

					(*n* = 38)	
Variable	Partial regression coefficient	Standardized Partial regression coefficient	T	*P*-value	95.0% confidence interval for B	
	(B)	(β)			Lower bound	Upper bound
Innate resilience	0.035	0.021	0.093	0.926	−0.737	0.808
Acquired resilience	−1.125	−0.382	−1.709	0.098	−2.469	0.219
Age (year)	−0.371	− 0.234	− 0.934	0.358	−1.183	0.440
Employment status (Dummy variable: Yes 1, No 0)	−12.148	−0.446	−2.648	0.013*	−21.515	−2.780
Age when diagnosed with SUD (year)	0.182	0.106	0.476	0.637	−0.598	0.961
Treatment time period (month)	0.040	0.224	1.034	0.310	−0.039	0.119
Abstinence period (month)	0.044	0.101	0.558	0.581	−0.117	0.205
R^2^ = 0.306, Adjusted R^2^ = 0.145						

*n* number of participants,

Statistical evaluation is performed by multiple regression analysis. *P < 0.05

Dependent variable is SRRS Stimulant Relapse Risk Scale (total)

## Discussion

Study participants had innate and acquired resilience scores of 35.3 ± 7.3 and 29.4 ± 4.7, respectively. This study identified the employment status and acquired resilience as two factors significantly associated with relapse risk.

First, our population had innate and acquired resilience scores of 35.3 ± 7.3 and 29.4 ± 4.7, respectively. A previous study using the same Bidimensional Resilience Scale observed respective scores of 38.2 ± 8.1 and 30.1 ± 5.5 in patients with AUDs [26]. While acquired resilience scores did not greatly differ between the two studies, our subjects’ innate resilience score was 2.9 points lower. A previous study reported low levels of resilience in individuals with a SUD [30]. This study found no significant difference in the association between relapse risk and innate or acquired resilience (Std. β = − 0.382, *P* < 0.05). However, there was a significant bivariate correlation showing that the higher the acquired resilience, the lower the relapse risk (*r* = − 0.314, *P* < 0.01). This suggests that resilience is enhanced as patients recover from SUDs. The idea of a resilience-related mechanism that protects people from psychological risk has empirical support, with findings demonstrating such a mechanism to reduce the effects of said risks, decrease negative chain reactions, and establish and maintain self-esteem and self-efficacy [15]. This indicates that interventions intended to instill self-esteem and self-efficacy in patients during their recovery should primarily involve modifying their environment, such as changing or avoiding interactions with others formerly associated with the SUD and correcting cognitive distortions [31]. Mindfulness’s efficacy in correcting cognitive distortions has been demonstrated in patients with SUDs [32]. Recent years have seen the application of workbook-based CBT based on evidence from the Matrix Model in the USA, with proven results [33].

Our findings, that the relapse risk was significantly and negatively correlated with innate and acquired resilience, with high acquired resilience predicting reduced relapse risk, illustrate the need for SUD recovery to focus on enhancing resilience. SUD prevention-related research on resilience has reported that such efforts to improve resilience need to be started in early childhood [34]. Moreover, resilience-related research has demonstrated the need for family-based interventions and social support, showing a community affinity-based intervention program to enhance resilience in families with teenagers—a demographic at high risk for alcohol and drug dependence [35]. This would necessitate programs to improve resilience starting in the teenage years. Moreover, resilience building requires a safe and stable treatment environment, where individuals can self-disclose with peace of mind. Other research has reported resilience to be enhanced through better self-disclosure by participation in meetings held in self-help groups and treatment centers aimed at preventing relapse in alcohol dependence [36].

Substance dependence must be treated using a dual approach—targeting withdrawal symptoms due to physical dependence as well as drug-seeking behavior due to mental dependence [37]. In addition, continuous therapy combining pharmacological with psychosocial interventions is essential for SUD recovery. We found that subjects with a comorbid mental illness were at a significantly lower relapse risk than those without (t [39] = 2.083, *P* < 0.05): Perhaps the fact that these individuals had already been receiving continued treatment is in part responsible.

We identified ongoing employment as a factor related to relapse risk. This finding could also be explained by subjects gaining employment as a result of reduced relapse risk. Employment is a form of self-actualization, which leads to improved self-esteem and self-efficacy. Employment can be equally conceived of as a desire to engage with the recovery process, a form of empowerment, a sign of taking personal responsibility, and as a significant role in someone’s life [38]. Employment history, marketable skills, and barriers have all been identified as essential elements of vocational assessments for patients with SUDs performed in SUD treatment programs [39]. Punishments for illegal drug use and difficulties with living impairment due to dependence can cause people to leave their jobs. However, employment’s association with acquired resilience shows the need for recovery programs to incorporate vocational support, tailored to a patient’s stage in the recovery process. Self-stigma has negative effects on self-esteem and self-efficacy and a continuing impact on wellbeing [40]. However, self-stigma can be reduced by attending a self-help group. In addition, it has been shown that outpatients with SUDs who attended a self-help group and also received psychotherapy had increased employment opportunities and an improved prognosis [41]. Although, for the sample in this study, there were no significant differences in how long participants had been attending a self-help group or in how many sessions they attended that affected acquired resilience and relapse risk; it appeared that attendance had a positive effect on self-esteem and self-efficacy and increased the chances of employment. Getting a job is not something that happens without social support. In SUD recovery support interventions, such as Individual Placement and Support [42], it is important in interactions with patients to promote acquired resilience by trusting the patient, believing in their potential and communicating the hope that they can work (general employment), even if they have a disability. This study showed, for the first time, that being employed may be related to the reduction of SUD relapse risk. Employment support services for individuals with SUDs should be reinforced as a community-based measure to support their recovery.

The sample is small; only 80 surveys were distributed because it is difficult to reach this population in large numbers. This study’s cross-sectional design prevents us from drawing inferences about the recovery process over time in individuals with SUDs. In addition, given the small sample size, our findings may not necessarily capture all facets of SUDs at the population level. Multiple regression analysis may have shown no significant difference between the regression risk and acquired resilience due to the small sample size. Furthermore, there may be individual differences in certain respects, such as how quickly and severely dependence forms, given the great variation in SUD, and, accordingly, their biological and psychoactive properties. Conducting a longitudinal study to shed light on how resilience changes during the recovery process is a task for future studies.

## Conclusion

Our findings provide some insight into the therapeutic benefits of recovery support of patients with SUD. Greater acquired resilience was correlated with lower relapse risks (*r* = − 0.314, *P* < 0.01); furthermore, reduced relapse risk was significantly associated with current employment (Std. β = − 0.446, *P* < 0.05).

The association of acquired resilience with employment status means that recovery programs need to incorporate employment support tailored to patients’ progress in the recovery process. Recovery support to enhance resilience in individuals with SUDs should include means to improve employment support services and ensure that patients continue treatment in the long term.

## Data Availability

The data analyzed in the current study are not publicly available due to confidential and potentially sensitive information. Data are available from the corresponding author on request.

## Abbreviations

AI: Anxiety and intention to use drug
AI: Awareness of illness
CBT: Cognitive–behavioral therapy
CD: Compulsivity for drug
EP: Emotionality problem
N: Number of participants
NE: Lack of negative expectancy for the drug
PL: Positive expectancies and lack of control over drug
SD: Standard deviation
SRRS: Stimulant Relapse Risk Scale
SUDs: Substance use disorders
